# Antiretroviral therapy outcomes among adolescents and young adults in a Tertiary hospital in Kenya

**DOI:** 10.4314/ahs.v21i1.2S

**Published:** 2021-05

**Authors:** Patrick Mburugu, Peter Muiruri, Nelly Opiyo, Justus Simba, Jane Adunda, Rosemary Kawira, Onesmus Gachuno

**Affiliations:** 1 Jomo Kenyatta University of Agriculture and Technology, Child Health and Pediatrics; 2 Kenyatta National Hospital, Kenya, Comprehensive Care Centre; 3 Jomo Kenyatta University of Agriculture and Technology, Biostatistics and Actuarial Sciences; 4 Jomo Kenyatta University of Agriculture and Technology, School of Nursing Sciences; 5 University of Nairobi, Department of Obstetrics and Gynecology

**Keywords:** Adolescents, HIV, Kenya, virologic suppression, young adults

## Abstract

**Background:**

Limited data is available on the treatment outcomes of HIV infected adolescents and young adults (AYA) in sub-Saharan Africa. HIV-infected adolescents and young adults (AYA) are at high risk of developing antiretroviral treatment failure.

**Objective:**

To determine the clinical, immunological and virologic outcomes of AYA at a tertiary hospital in Kenya.

**Methodology:**

A longitudinal study was conducted among AYA age 10–24 years attending Kenyatta National Hospital comprehensive care center. Clinical data was abstracted from electronic medical records for study participants with at least 6 months of follow-up using a structured data abstraction sheet.

**Results:**

A total of 250 AYA age 10 to 24 years were included. The median age was 16 years. The median CD4 cell count was 650.6 cells/mm3 (IQR 350.7–884.0). More than half (60.6%) of AYA had a CD4 cell count higher than 500 cells/mm^3^. Overall, 76.9% of AYA had achieved viral suppression (viral load <1000 copies/ml). There was a significant increase in virologic failure with higher age and late adolescents and young adults were more likely to have a viral load > 1000 copies/ml p<0.012.

**Conclusion:**

The overall virologic suppression in this cohort of AYA was sub-optimal. Both immunological and virologic outcomes were worse among late adolescents (18–19 years) and young adults (20–24 years).

## Introduction

Adolescents and young adults (AYA) represent an increasing proportion of people living with HIV in the world. Over 1.6 million adolescents age 10–19 and 5 million young people age 15–24 are living with HIV 1. Out of these 1.5 million (89%) HIV infected adolescents live in sub-Saharan Africa. Adolescents have low levels of testing, linkage and retention to care and poor adherence to antiretroviral treatment 2,3. Consequently clinical, immunological and virologic outcomes in adolescents have consistently shown poor results 4. The number of AIDS-related deaths among adolescents tripled between the year 2000 and 2015 5.

Adolescents and young adults are at high risk of developing treatment failure and drug resistance, which has dire consequences in low resource settings such as Kenya, where there are limited choices of antiretroviral regimens. Across sub-Saharan Africa, many children who were infected at birth are now becoming adolescents. The number of adolescents and young adults on antiretroviral treatment has been increasing reflecting treatment success of HIV infected children 6.

AYA have unique characteristics that predisposes them to treatment failure and drug resistance such as poor insight into the disease and treatment 7. This makes close follow up and frequent assessment of treatment outcomes necessary. The existing literature on improving treatment outcomes is mainly focused on children and adults, and very limited in regards to AYA. Despite major advances in treatment and access to antiretroviral treatment, little is known about treatment outcomes in this age group. The study assessed the treatment outcomes of AYA actively on follow up at a tertiary hospital setting to determine their clinical, immunological and virologic outcomes.

## Methods

### Study Design

A longitudinal study was conducted as part of an interventional study to determine the effect of mobile phone calls on retention to care among adolescents and young adults on care and treatment at Kenyatta National Hospital Comprehensive Care Clinic between January 2017 and November 2017. A review of medical records was performed to determine the clinical, immunological and virologic outcomes in AYA 10 to 24 years on active follow.

### Study Site

The study was carried out at Kenyatta National Hospital Comprehensive Care Centre which at the time of the study had 9,915 active HIV patients on care and treatment, among them 362 adolescents age 15–19 years and 693 children between 0–14 years. Patient's data is entered into an electronic medical records database (IQ care) during routine clinic visits. Data on clinical variables, outcomes, treatment regimens and laboratory records were abstracted from the electronic medical records data which is routinely updated at each clinic visit. AYA are typically seen in the clinic monthly or every three months for clinical monitoring, with a bi-annual viral loads and CD4 cell count monitoring.

### Study Outcomes

The main study outcomes were improvement in WHO staging, immunological status and virologic failure. Immunological failure was defined as CD4 cell count < 500 cells/mm^3^ and virologic failure as a viral load of > 1000 copies/ml.

### IRB Approval

The study was approved by Kenyatta National Hospital/University of Nairobi Ethical Review Committee (ERC) and University of Washington Institutional Review Board.

### Data Management and Statistical Analysis

Data were collected and entered into a password-protected MS-Access database. The database was set within required limits and restrictions to reduce errors. Exploratory data analysis was carried out to identify extreme values and inconsistencies. On completion of the data entry, the data was exported into SPSS Version 21.0 for analysis.

Continuous values were summarized as means, medians, standard deviation and interquartile ranges. Discrete values were summarized using frequencies and percentages. Categorical variables were compared using chi-square test and Fishers exact test. Continuous variables were analyzed using Student t-test and Mann Whitney test by comparing means. A P value <0.05 was considered significant. The associations between social demographics characteristics and treatment outcomes were measured by Odds Ratios (OR).

## Results

### Characteristics of the Study Population

A total of 250 AYA age 10 to 24 years were included with a median age of 16 years. Of those in adolescence, 71 (28.4 %), 90 (36%) and 59 (23.6%) were in early (10–13 years), mid (14–16 years) and late adolescence (18–19 years) respectively, while 30 (12%) were young adults (20–24 years). Ratio of males 124 (49.6%) vs females 126 (50.4%) was almost 1. Majority of the study participants 226 (90.4%) had a parent as the primary caregiver while the rest had guardians. Almost half; 120 (48.2%) attended clinics unaccompanied, while (90) 36.1%, 23 (9.2%), (6) 2.4% 10 (4.0%) were accompanied by their mothers, fathers, siblings and guardians respectively. About two thirds 169 (67.6%) attended all clinics as scheduled while a third 81( 32.4%) missed their scheduled clinics. There was no statistical difference on attendance of scheduled clinic visits among the various age groups (p=0.764).

### Treatment History

All AYA in this study were on antiretroviral therapy (ART). The majority of 173 (69.2%) were on a 1^st^ line antiretroviral regimen, while 77 (30.8%) were on their 2^nd^ line regimen as per the national guidelines in Kenya. The most frequent ART regimen combination at ART initiation comprised of two nucleoside reverse transcriptase inhibitors (NRTIs); [zidovudine or abacavir or tenofovir or stavudine] plus lamivudine and one non-nucleoside reverse transcriptase inhibitor (NNRTI), nevirapine or efavirenz. Only a third ( 33 (13.2%) were initiated on a protease inhibitor-based regimen. On the current ART regimen; majority 171 (68.4%) were on two nucleoside reverse transcriptase inhibitors (NRTIs); [zidovudine or abacavir or tenofovir plus lamivudine] and one non-nucleoside reverse transcriptase inhibitor (NNRTI), [nevirapine or efavirenz]. Seventy seven (30.8%) were on a protease inhibitor-based regimen while two (0.8%) were on an integrase inhibitor-based regimen. (This is shown in Table 1). A review of records revealed that 85 (34.0%) of study participants had a history of ART substitution.

**Table 1:** Antiretroviral Therapy Regimen at Kenyatta National Hospital Comprehensive Care Centre, Kenya 2017

**Initiation regimen**	**n(%)**
D4T/3TC/NVP	1 (0.4)
TDF/3TC/NVP	2 (0.8)
AZT/3TC/NVP	47(18.8)
ABC/3TC/NVP	11 (4.4)
TDF/3TC/LPVr	8 (3.2)
AZT/3TC/LPVr	7 (2.8)
D4T/3TC/EFV	4(1.6)
TDF/3TC/EFV	37(14.8)
AZT/3TC/EFV	63 (25.2)
ABC/3TC/EFV	50 (20)
TDF/3TC/ATVr	3(1.2)
ABC/3TC/LPVr	15 (6.0)
RAL/ATVr	1 (0.4)
TDF/3TC/DTG	1 (0.4)
**Current regimen**	**n(%)**
TDF/3TC/NVP	3(1.2)
AZT/3TC/NVP	32(12.8)
ABC/3 TC/NVP	8 (3.2)
TDF/3TC/LPVr	14 (5.6)
AZT/3TC/LPVr	12 (4.8)
TDF/3TC/EFV	67 (26.8)
AZT/3TC/EFV	43 (17.2)
ABC/3 TC/EFV	18 (7.2)
TDF/3TC/ATVr	29(11.6)
AZT/3TC/ATVr	3(1.2)
ABC/3 TC/LPVr	15 (6.0)
ABC/3 TC/ATVr	1 (0.4)
RAL/3 TC/LPVr	1 (0.4)
RAL/3TC/DRV/RTV	1 (0.4)
RAL/ATVr	1 (0.4)
TDF/3TC/DTG	2 (0.8)

### Treatment Outcomes

Treatment outcomes 6 months after enrollment into the study showed that the mean body weight (SD) significantly increased from 46.3 (12.7) to 50.5 (11.1) p<0.001, the WHO clinical staging significantly deteriorated from stage I towards stage IV p=0.003, there was no significant change in CD4 cell count ( >= 500 cells/mmm^3^ Vs. <500 cells/mm^3^) p=1.000 and no significant changes in viral loads (<1000 copies/ml Vs. >= 1000 copies/ml) p=1.000. (The results are shown in Table 2)

**Table 2:** Comparison of Treatment Outcomes at Enrollment and at 6 Months, Kenyatta National Hospital Comprehensive Care Centre, Kenya 2017

Variable	Enrolment	At 6 months	P value
**Weight**			
Mean (SD)	46.3 (12.7)	50.5 (11.1)	<0.001
**WHO clinical stage**			
I	215 (86.0)	193 (79.4)	0.003
II	19 (7.6)	28 (11.5)	
III	14 (5.6)	19 (7.8)	
IV	2 (0.8)	3 (1.2)	
**CD4 cells count/mm^3^**			
>=500	148 (60.9)	143 (60.6)	1.000
<500	95 (39.1)	93 (39.4)	
**Viral load copies/ml**			
<1000	188 (75.2)	186 (76.9)	1.000
>=1000	62 (24.8)	56 (23.1)	

### WHO Clinical Staging

Overall at 6 months of the study a larger proportion of AYA were in WHO clinical stage I 193 (79.4%), 28 (11.5%) were in WHO clinical stage II, 19 (7.8%) in WHO clinical stage III and 3 (1.2) in WHO clinical stage IV. At 6 months of the study there was no significant association in improvement in WHO clinical staging depending on gender, adolescent age group or the primary caregiver. Although not statistically significant, AYA who missed clinic appointments were more likely to move to an advanced WHO stage compared to those who did not miss appointments [OR 1.7(95% CI 0.6–4.8), OR 1.0 (95% CI), p=0.304]. Those that had their regimen substituted were more likely to be in an advanced WHO clinical stage compared to those that were not OR 0.3 (95% CI 0.1–0.8), 1.0 (CI 95%) p=0.01. (The results are summarized in Table 3).

**Table 3:** Factors associated with WHO clinical staging at 6 months, Kenyatta National Hospital Comprehensive Care Centre, Kenya 2017

Variable	WHO clinical stage at 6 months		OR (95% CI)	P value
	I & II	III & IV		
**Gender**				
Male	106 (48.0)	14 (63.6)	0.5 (0.2–1.3)	0.161
Female	115 (52.0)	8 (36.4)	1.0	
**Age**				
10–13	66 (30.0)	0		0.009
14–17	83 (37.7)	10 (47.6)	1.0	
18–19	45 (20.5)	6 (28.6)	0.9 (0.3–2.6)	0.853
20–24	26 (11.8)	5 (23.8)	0.6 (0.2–2.0)	0.427
**Patient primary caregiver**				
Parents	197 (90.0)	22 (100.0)	-	0.236
Guardian	22 (10.0)	0		
**Missed appointment**				
Yes	74 (33.5)	5 (22.7)	1.7 (0.6–4.8)	0.304
No	147 (66.5)	17 (77.3)	1.0	
**Regimen substituted**				
Yes	70 (31.7)	13 (59.1)	0.3 (0.1–0.8)	0.010
No	151 (68.3)	9 (40.9)	1.0	

### Immunological Outcomes

At 6 months of the study the median CD4 cell count for all age categories was 650.6 cells/mm^3^ (IQR 350.7–884.0). Considering all age categories, more than half (60.6%) of AYA had a CD4 cell count with more than 500 cells/mm^3^. There was a decrease in CD4 cell count with higher age, with 92.3 % of early adolescents (10–13 years), 62.2 % of mid adolescents (14–17 years), 38.8 % of late adolescents (18–19 years) and only 23.3 % of young adults having a CD4 cell count above 500 cells/mm^3^ p<0.001. (The results are shown in Table 4).

**Table 4:** Trends in CD4+ Count and Viral Loads among AYA age Categories, Kenyatta National Hospital Comprehensive Care Centre, Kenya 2017

**Variable**	**CD4 cells at 6 months**		**P value**
	**>=500 cells/mm^3^**	**<500 cells/mm^3^**	
**Age**			
10–13	60 (92.3)	5 (7.7)	<0.001
14–17	56 (62.2)	34 (37.8)	
18–19	19 (38.8)	30 (61.2)	
20–24	7 (23.3)	23 (76.7)	
**Variable**	**VL at 6 months**		**P value**
	**<1000** **copies/ml**	**>=1000 copies/ml**	
**Age**			
10–13	58 (87.9)	8 (12.1)	0.012
14–17	73 (78.5)	20 (21.5)	
18–19	34 (68.0)	16 (32.0)	
20–24	19 (61.3)	12 (38.7)	

On factors associated with CD4 cell count at 6 months, early adolescents were more likely to have a CD4 cell count more than 500 cells/mm^3^ (OR 7.3 (2.7–19.9 p<0.001) while young adults were more likely to have a CD4 cell count of less than 500 cells/mm3 (OR 0.2 (0.1–0.5 p<0.001). (The results are shown in Table 5).

**Table 5:** Factors associated with CD4+ Count Level at 6 months, Kenyatta National Hospital Comprehensive Care Centre, Kenya 2017

Variable	CD4 at 6 months		OR (95% CI)	P value
	>=500 cells/mm^3^	<500 cells/mm^3^		
**Gender**				
Male	71 (49.7)	44 (47.3)	1.1 (0.7–1.9)	0.725
Female	72 (50.3)	49 (52.7)	1.0	
**Age**				
10–13	60 (42.3)	5 (5.4)	7.3 (2.7–19.9)	<0.001
14–17	56 (39.4)	34 (37.0)	1.0	
18–19	19 (13.4)	30 (32.6)	0.4 (0.2–0.8)	0.008
20–24	7 (4.9)	23 (25.0)	0.2 (0.1–0.5)	<0.001
**Patient primary caregiver**				
Parents	129 (90.8)	84 (90.3)	0.9 (0.4–2.4)	0.904
Guardian	13 (9.2)	8 (8.7)	1.0	
**Missed appointment**				
Yes	49 (34.3)	28 (30.1)	1.2 (0.7–2.1)	0.506
No	94 (65.7)	65 (69.9)	1.0	
**Regimen substituted**				
Yes	38 (26.6)	45 (48.4)	0.4 (0.2–0.7)	0.001
No	105 (73.4)	48 (51.6)	1.0	

### Virolog ic Failure

At a reference point of 6 months ( 186 ) 76.9% of adolescents and young adults had a viral load <1000 copies/ml, while 93 (39.4%) had a viral load of >1000 copies/ml. There was a significant likelihood of virologic failure with higher age; with young adults more likely to have a viral load > 1000 copies/ml p<0.012. (The results are shown in Table 4). Although not statistically significant early adolescents were twice as likely to have virologic suppression compared to other age categories in the study OR 2.0 (0.8–4.8) p=0.126. (The results are shown in Table 6).

**Table 6:** Factors associated with Viral Load suppression at 6 months, Kenyatta National Hospital Comprehensive Care Centre, Kenya 2017

Variable	VL at 6 months		OR (95% CI)	P value
	<1000 copies/ml	>=1000 copies/ml		
**Gender**				
Male	87 (46.8)	32 (57.1)	0.7 (0.4–1.2)	0.174
Female	99 (53.2)	24 (42.9)		
**Age**				
10–13	58 (31.5)	8 (14.3)	2.0 (0.8–4.8)	0.126
14–17	73 (39.7)	20 (35.7)	1.0	
18–19	34 (18.5)	16 (28.6)	0.6 (0.3–1.3)	0.168
20–24	19 (10.3)	12 (21.4)	0.4 (0.2–1.0)	0.058
**Patient primary caregiver**				
Parents	169 (91.4)	49 (89.1)	1.3 (0.5–3.5)	0.610
Guardian	16 (8.6)	6 (10.9)	1.0	
**Missed appointment**				
Yes	64 (33.9)	16 (28.6)	1.3 (0.7–2.5)	0.458
No	123 (66.1)	40 (71.4)	1.0	
**Regimen substituted**				
Yes	62 (31.2)	18 (50.0)	0.8 (0.4–1.4)	0.370
No	137 (68.8)	18 (50.0)	1.0	

## Discussion

Our study findings demonstrated that 76.9% of AYA in this cohort had achieved viral load suppression. Our study cohort viral load suppression was lower than the national average reported rate of 88.4% for patients 0–64 years 8. The viral load suppression is slightly higher than the 67.1% found by KENPHIA among children aged 0–14 years but much lower than the 90.6% among those 15–64 years of age. The relatively low rates of viral load suppression in AYA has been revealed in several other studies^9^,^10^. In our study, young adults were less likely to achieve adequate virologic suppression, followed by late adolescents. Majority of early adolescents (87.9%) had achieved virologic suppression.

Median CD4 cell count was 650 cells/mm^3^, with 60% of adolescents having a CD4 cell count > 500 cells/mm^3^. This immunological response is consistent to study findings in Asia and South Africa where the median CD4 cell count was fond to be 657cells/mm^3^ and 686 cells/mm^3^ respectively^11^,^12^. A similar trend of higher virologic suppression among early adolescents compared to mid, late adolescents and young adults was observed with CD4 cell count, where majority of early adolescents had a better immunological outcome compared to the other age groups. This might be explained by parental oversight to treatment for early adolescents. More than half of late adolescents and young adults had CD4 cell count of < 500 cells/mm^3^. This trend of a decrease in CD4 cell count and increased chances of virologic failure with advancing age was observed in a study by Mburugu and colleagues (2016) where mid and late adolescents had poor immunologic, virologic outcomes and poor adherence to treatment^13^. Similarly Geoffrey and colleagues^14^ documented in a baseline characteristics study in South Africa that mid and late adolescents had lower median CD4 cell counts ^17^. In the same study viral suppression was poorest in mid adolescents at any point time of ART. The poor immunological and virologic outcomes could be explained by less adherence support during transition to adult HIV programs leading to poor adherence to ART, less support from parents and guardians and complexities associated with the developmental stages of adolescence and young adulthood.

At our last assessment at 6 months, a larger proportion of AYA in our study (79.4%) were in WHO clinical stage I, while 11.5%,7.8% and 1.2 % were in WHO clinical stage II, III and IV respectively. Thus majority of the adolescents were stable with absence of recent opportunistic infections. However on further analysis, during the six months study period, there was significant deterioration in the clinical staging from WHO clinical stage I to IV. The finding of having a majority of HIV infected adolescents being stable was also found in a longitudinal study by Allison et al where they found 85% of adolescents in there cohort to be stable. ^18^

## Limitations

This was a cross-sectional study that utilized routinely collected clinical outcome data from an electronic medical records system as part of a clinical trial. The study involved a single site which is a tertiary teaching hospital thus the results may have limited generalizability. However this study provides an insight into the treatment outcomes of HIV infected adolescents and identifies the age categories that need special attention during care and treatment for that age group.

## Conclusion

Viral load suppression in this cohort of adolescents was suboptimal with late adolescents (18–19 years) and young adults (20–24 years) being more likely to have virologic failure. Interventions in this program should targets adolescents and young adults with special focus to late adolescents and young adults to improve treatment outcomes.
